# Supplementary material for: Prognostic value of neutrophil-lymphocyte ratio in gastroenteropancreatic neuroendocrine neoplasm: a systematic review and meta-analysis
Source: PeerJ. 2025 Apr 7;13:e19186. doi: 10.7717/peerj.19186 (PMC11984474; doi:10.7717/peerj.19186)
Supplement: Supplemental Information 1 [file peerj-13-19186-s001.docx]

**Embase:** ('neuroendocrine tumor'/exp OR 'neuroendocrine tumors'/exp OR 'tumor, neuroendocrine' OR 'tumors, neuroendocrine') AND ('neutrophil to lymphocyte ratio'/exp OR 'neutrophil-to-lymphocyte ratio'/exp OR 'neutrophil/lymphocyte ratio'/exp OR 'NLR'). **PubMed:** ((Neuroendocrine Tumors) OR (Neuroendocrine Tumor) OR (Tumor, Neuroendocrine) OR (Tumors, Neuroendocrine)) AND ("neutrophil lymphocyte ratio" or "neutrophil to lymphocyte ratio" or "neutrophil-to-lymphocyte ratio" or "neutrophil/lymphocyte ratio" or "NLR"). **Web of science:** ((AB= (Neuroendocrine Tumors) OR AB= (Neuroendocrine Tumor) OR AB= (Tumor, Neuroendocrine) OR AB= (Tumors, Neuroendocrine)) AND ((AB= (neutrophil-lymphocyte ratio)) OR AB=(NLR)) OR AB= (neutrophil-to-lymphocyte ratio).
