# Supplementary material for: Prognostic value of neutrophil-lymphocyte ratio in gastroenteropancreatic neuroendocrine neoplasm: a systematic review and meta-analysis
Source: PeerJ. 2025 Apr 7;13:e19186. doi: 10.7717/peerj.19186 (PMC11984474; doi:10.7717/peerj.19186)
Supplement: Supplemental Information 2 [file peerj-13-19186-s002.docx]

The rationale for exploring the correlation between Pretreatment NLR and prognosis of gastroenteropancreatic neuroendocrine neoplasm (GEP-NEN) is that inflammatory response played a decisive role in different stages of tumor development, including initiating, promoting, malignant transformation, invasion, and metastasis^1^. On the one hand, tumors change their microenvironment by secreting a variety of cytokines, chemokines to weaken the systemic immune response and promotes tumorigenesis and progression; on the other hand, systemic and local tissues are also infiltrated by immune cells and cytokine secretion to alter the tumor microenvironment and kill tumor cells.^2^ The NLR, as the ratio of neutrophils to lymphocytes in the peripheral blood, not only reflects the systemic tumor-associated inflammatory response, but may also reflect bone marrow versus lymph, innate versus adaptive immunity, chronic inflammation versus acute immune rejection, tumor and antitumor immune equilibrium^3^. However, the predictive role of NLR in GEP-NEN remains controversial.

1. Grivennikov SI, Greten FR, Karin M. Immunity, inflammation, and cancer. *Cell.* 2010;140(6):883-899.

2. Hinshaw DC, Shevde LA. The Tumor Microenvironment Innately Modulates Cancer Progression. *Cancer research.* 2019;79(18):4557-4566.

3. Park W, Lopes G. Perspectives: Neutrophil-to-lymphocyte Ratio as a Potential Biomarker in Immune Checkpoint Inhibitor for Non-Small-Cell Lung Cancer. *Clinical lung cancer.* 2019;20(3):143-147.
